# Exploring the potential of the COI gene marker for DNA barcoding of planktonic foraminifera

**DOI:** 10.1038/s41598-025-03842-7

**Published:** 2025-06-01

**Authors:** Ana Carolina Bercini Gusmao, Robin L. van Dijk, Elsa B. Girard, Katja T. C. A. Peijnenburg, Jan Macher, Michal Kucera, Raphaël Morard

**Affiliations:** 1 Max Planck Institute for Marine Microbiology, Bremen, Germany; 2 Naturalis Biodiversity Center, Leiden, The Netherlands; 3 Department of Environmental Biology, Institute of Environmental Sciences (CML), Leiden University, Leiden, The Netherlands; 4 Department of Freshwater and Marine Ecology, Institute for Biodiversity and Ecosystem Dynamics, University of Amsterdam, Amsterdam, The Netherlands; 5 MARUM Center for Marine Environmental Sciences, University of Bremen, Leobener Strasse, 28359 Bremen, Germany

**Keywords:** Planktonic foraminifera, COI, SSU, qPCR, Metabarcoding, Biological techniques, Ecology, Microbiology, Molecular biology, Ocean sciences

## Abstract

Metabarcoding is a cornerstone of modern ecology, but its accuracy is dependent on the chosen gene marker. While the small subunit ribosomal DNA (SSU) is a powerful tool to describe protist diversity, its reliability in retrieving the composition of communities is less obvious. It is particularly challenging to obtain quantitative estimates of abundance in planktonic foraminifera, where the variability of the SSU gene copy number can span three orders of magnitude. As an alternative, we explored the potential of the mitochondrial cytochrome c oxidase subunit I (COI) marker. We developed a reference barcode library of 130 sequences of a 1200 bp long COI fragment belonging to 26 morphospecies of foraminifera and performed 201 single-cell qPCR quantifications to evaluate the relationship between the number of COI copies, and the size of individual foraminifera. We found that the COI evolves between 25 and 1000 times slower than the SSU and therefore has a poor taxonomic resolution. However, we observed a significant relationship between COI copy number and foraminifera size. These results suggest that SSU and COI can play complementary roles: the SSU is well-suited for capturing taxonomic diversity, while the COI is useful to retrieve crude information on the community composition.

## Introduction

Metabarcoding is a powerful tool to describe microbial eukaryotic communities^1^. It provides a list of short sequences (or barcodes), associated with occurrences in the dataset to accurately describe the taxonomic composition of biological communities. The adequacy of metabarcoding to describe actual communities hinges on two notable properties of the selected gene markers. First, the taxonomic resolution of the marker should ideally resolve the species level, meaning that this region of the DNA evolves fast enough and produce a barcode gap. A barcode gap is observed when the divergence of a given gene among organisms belonging to the same species is smaller than divergence among organisms from different species^2,3^. Second, the chosen marker should scale to the biomass of the studied taxa to represent their proportionality in the DNA template^3^. When the chosen marker does not fulfill either of these conditions, it results in a distorted description of living communities.

The small subunit of the ribosomal RNA gene (SSU) is a genetic marker that^4^ served to build worldwide inventories of protist diversity in marine^5^ and terrestrial environments^4^. A general relationship exists between the size of organisms and the number of SSU copies per cell/individual when considering size ranges covering 10–1000 µm in phylogenetically diverse groups^6^. For example, the diatom *Thalassioria weisflogii* with cells of 10 µm harbor ~ 49 SSU copies per cell^7^, the collodaria *Sphaerozoum fuscum* with cells of 112 µm harbor ~ 41,000 SSU copies per cell^8^ while the ciliate *Tintinnopsis sp.* with cells of ~ 195 µm have ~ 120,000 SSU copies per cell^9^. However, this relationship is less obvious when considering the scale of size variability within a single taxon or clade. This is the case for foraminifera, where the number of SSU copies varies between 100 and 300,000 gene copies^10^ for similar sized specimens from the species *Neogloboquadrina pachyderma*^10,11^, and no correlation between size and gene copy number existed in five species tested^10^. This questions the accuracy of metabarcoding dataset to accurately represent communities of organisms^12,13^.

As an alternative barcode to SSU, the cytochrome c oxidase I (COI) has been sequenced for 17 phylogenetically distant species of benthic foraminifera by Macher et al. (2015), revealing strong congruence between SSU- and COI-based phylogenetic trees. This study demonstrated that the ~ 310 bp COI barcode enables species identification of benthic foraminifera. Additionally, COI showed lower intra-genomic variability than SSU and higher amplification success in a survey of 200 specimens from 22 morphospecies^10^, making the COI less prone to diversity estimate inflation. The reliability of the COI barcode in retrieving species composition using the metabarcoding approach was tested on mock communities and environmental samples of coral reef sediment samples^11^, and environmental samples of beach transect in the Netherlands^14^, and showed a recovery of 90% of foraminifera sequences, detection of all but a single species in the mock communities, and clear structuring of the dataset along environmental gradients in both studies. Lastly, single-cell qPCR was used to determine if a correlation exist between number of COI copies and size of 193 specimens of seven species of Larger Benthic Foraminifera (LBF)^13^, which are symbiont-bearing species of foraminifera that are phylogenetically unrelated, morphologically diverse and can reach several mm in size and are major carbon producers in reef environments. Significant, but species-specific relationships existed between size and COI copy number of the seven species analyzed. Specific calibration were applied on metabarcoded samples that lead to an estimation from proportion of relative abundance with a difference of ± 5% on average from counted environmental samples. Altogether, these results opens pathways for accurate community description via metabarcoding but so far all studies have been conducted on genetically distant benthic foraminifera, and only a single species of planktonic foraminifera has been sequenced so far^15^.

Here we explore the potential of COI as a barcode for planktonic foraminifera, by first establishing a reference barcode library covering the diversity of the three main clades of planktonic foraminifera, the Spinose, Non-spinose, and Microperforate clades, to evaluate the taxonomic resolution of COI compared to the SSU marker, and second, by assessing if a relationship exists between the size of individual foraminifera cells and the number of gene copies using quantitative PCR. Nearly all species of planktonic foraminifera have been barcoded for the SSU^16^ allowing to test the discrimination power of the COI marker for phylogenetically close species. Also, we use and complement a collection of 119 single-cell specimens where the number of SSU gene copy number was measured in a previous study^17^, allowing a direct comparison between the number of COI and SSU copies number which has not been done so far. Finally, we integrate the benthic and planktonic foraminifera single-cell quantification results to assess if a global relationship exists across the entire phylum^16–20^.

## Material and methods

### Sample collection and database assembly

In total, we used 311 specimens for the entire study, collected during 10 cruises at 38 stations and belonging to 26 planktonic foraminifera species. We assembled a dataset by combining new sample collection, re-use of existing DNA extraction collection, and published data for the study. The novel samples used in this study were collected during a cruise on *RV Pelagia* (64PE513) in the South Atlantic at a unique station (98928S, 20313W) on 22.02.2023. The samples were collected using a multinet with a mesh size of 200 µm between 0 and 300 m depth. The full zooplankton sample was fixed in 99% EtOH, which was replaced once within 24 h, and stored at − 20 °C until further processing. Single cell foraminifera were sorted under a stereomicroscope at Naturalis Biodiversity Center (Leiden, the Netherlands) and identified following the taxonomy of Brummer and Kucera^18^. Selected specimens were imaged using a Zeiss V20 stacking stereomicroscope with Axiovision software (Zeiss, Germany). Hereafter, they were transferred to GITC* extraction buffer, frozen at − 80 °C until processing in the lab where the DNA was extracted following the GITC* procedure as explained in Weiner et al.^19^.

Next, available DNA extractions in the collection of the University of Bremen were selected for the study. These samples were extracted to measure the number of SSU copies in foraminifera in 2020^17^ using the DOC DNA extraction protocol^19^ where the single-cell foraminifera were entirely dissolved into 50 µl of DOC and were stored at 4 °C since extraction. Less than four years elapsed between the extraction and the generation of data of the present study. Whilst it could be expected that long storage in DOC buffer would affect the quality of the extract, the effect of long-term storage of single-cell foraminifera in DNA extraction buffer on PCR success has been tested up to 10 years after the DNA extraction procedure was completed, and there was no evidence that it impacts the DNA preservation negatively^19^. We utilized 204 DOC DNA extraction including 119 where SSU gene copy number was quantified previously^17^. Prior to DNA extraction, each specimen was photographed using a KEYENCE VHX 6000 digital microscope in a standard position to produce focus-stacked 2.5D images, and individual cell volumes were quantified as in Millivojevic et al. (2021). Finally, the collection was completed with existing GITC* DNA extraction used in Morard et al. (2019) and older DOC extraction to increase the taxonomic coverage of the present study.

The collection details and taxonomic identification of every single specimen used for reference barcoding are detailed in Supplementary Material 1 and those used for gene copy quantification in Supplementary Material 2.

### Barcode library

To amplify a fragment of ~ 1200 bp of the foraminifera COI, we designed a new primer pair Macher_COI_long_Rotaliida_f (5′–GGATTAATTGGAGGATCAATTGG–3′) and Macher_COI_long_Rotaliida_r (5′–CATAGATWCGTCTAGGAAAACC–3′) based on the full-length COI genes of Foraminifera^21^. As COI fragments were amplified in Bremen and Leiden laboratories, we used two different in-house protocols with the same primers. In Bremen, the PCR amplification was carried out by mixing 1 µl of DNA extract with 0.4 µM of each primer, 3% of DMSO, 1X HF Thermo Fisher buffer, 2.5 µM of MgCl2, 0.2 µM of dNTP, and 0.3 units of polymerase in a final volume of 15 µl. PCR amplification conditions were as follows: initial denaturation at 98 °C for 30 s followed by 35 cycles at 98 °C for 10 s, 65 °C for 30 s and 72 °C for 30 s, and 2 min of final extension at 72 °C. The resulting positive PCR products were purified using the QIAquick PCR purification kit (QIAGEN) and directly sequenced by an external provider (LGC Genomics, Berlin). In Leiden, the PCR amplification was carried out by mixing 1 µl of DNA extract with 0.2 µM of each M13 tailed primer, 3% DSMO, 1X Phusion HF Thermo Fisher buffer, 2.5 mM MgCl_2_, 0.05 mM of dNTPs, 0.3 units of Taq polymerase, and 8.8 µl Ultrapure MilliQ water was added to come to a final volume of 15 µl. PCR amplification conditions were as follows: initial denaturation at 98 °C for 30 s followed by 40 cycles at 98 °C for 10 s, 55 °C for 30 s and 72 °C for 30 s, and 10 min of final extension at 72 °C. The resulting positive PCR products were sequenced at BaseClear B.V. for Sanger sequencing (Leiden, the Netherlands).

The obtained chromatograms were manually checked, complementary fragments of the same sequence were de novo assembled, primer sequences were removed from both ends, and consensus sequences were deposited on NCBI under the accession number PQ626421-PQ626432 and PQ676554-PQ676671 and provided in the Supplementary Material 1.

### SSU versus COI barcode resolution

We used a parallel approach to compare the SSU and COI barcode taxonomic resolution within and between the three main clades of planktonic foraminifera. First, we only compared the number of identical sites between alignments of SSU and COI sequences at increasing taxonomic levels as a crude measurement of the genetic divergence. We used all unique complete COI sequences generated in this study (n = 41), and for the SSU, we used the 356 reference sequences representing the entire documented diversity to date^16^. Then each set of sequences was aligned automatically using MAFFT v.7^22^, and the proportion of identical sites between each sequence was calculated. We then plotted the intra-morphospecies distance as a measure of the intra-genomic and cryptic diversity variability, the inter-species distance as a measure of the genetic distance between sister species within the same genus, the inter-genus distances as a measure of genetic distance between genera of the same clade and finally the inter-clade distance, and compared the pairwise distances using a *t*-test.

Second, we used phylogenetic inferences to calculate the evolutionary distances between morphological species. We selected a single representative sequence for all species that have been sequenced for both SSU and COI, aligned the two sets of sequences automatically with MAFFT and calculated the phylogenetic inferences with PhyML^23^ using the Smart Model Selection option to choose the best model of evolution^24^, and assessed the topology robustness with 1000 transfer bootstrap^25^ and plotted the topologies with iTOL^26^. Next, we calculated the patristic distances for both trees and compared the SSU and COI rate of evolution using the same pairwise categories as to measure the different evolutionary rates between markers.

### Production of qPCR standards for the measurement COI copy number per individual

To produce standard curves for qPCR assays on multiple species per clade, we PCR amplified a COI fragment that we quantified accurately to then produce standard curves. The fragment has been identified by Macher et al.^15^ who developed degenerated primers to amplify all benthic foraminifera clades. We modified the primer to have a non-degenerated sequence that would amplify only the planktonic foraminifera that belong to the clade Rotaliida^27^. We defined an alternative version of the primer as Pforam COI-Fwd (5′–GTGGTGTTAATGCTGGTTGAAC–3′) and Pforam COI-Rev (5′–AAACTTCTGGATGTCTAAGAAATC–3′). We amplified the fragment necessary to produce the standard curves by PCR using the species *Trilobatus sacculifer*, *Neogloboquadrina dutertrei,* and *Globigerinita glutinata*, belonging to the clades Spinose, Non-spinose, and Microperforate, respectively. The master mix for each PCR reaction (final volume 15 µl) was composed of 8.7 µl of RNA-free water, 3 µl of Green Buffer (1X µmol/l concentration), 0.3 µl for each primer in 10 µM concentration, 0.75 µl MgCl^2^ in 50 mM concentration, 0.45 µl of 100% DMSO, 0.3 µl of DNTP mix, 0.15 µl of polymerase Phusion Green Hot Start II HF (2U/µl) and 1 µl of DNA template. The cycling conditions were 98 °C for 30 and 10 s for denaturation, 60 °C for 30 s for annealing, and 72 °C for 30 s, and 10 min for extension in 40 cycles. The success of PCR amplifications was checked by gel electrophoresis and the PCR purification was done using the QIAquick purification kit following the manufacturer’s instructions.

### qPCR assays of the single-cell DNA extracts

The DNA concentration of two specimens from each species was independently measured five times, with 1 µl of each PCR purified product and using the Promega QuantiFluor dsDNA System commercial kit for Quantus Fluorescence following the manufacturer’s instructions. We created one standard curve for each specimen (2 per species) by a tenfold serial dilution from 10^−1^ to 10^−8^. The 10^−1^ and 10^−2^ dilutions were excluded for the subsequent qPCR reactions as their concentrations were too concentrated to be relevant for the qPCR measurements. We calculated the number of COI copies of single-cell foraminifera using the average of replicates (ng/µl), following Eq. 1.

1 $${\text{Molecules}}/\upmu {\text{l}} = \left( {{\text{DNA concentration}}\left( {{\text{g}}/\upmu {\text{l}}} \right)} \right)/\left( {{\text{Fragment length}} \times 660} \right) \times 6.022 \times 10^{23}$$

To determine the stability and consistency of the results, we first implemented a cross-design analysis of standard curves. This approach evaluated the need of species-specific standard curves by comparing results from three representative species. To achieve this, we performed three qPCR reactions, each composed by a negative control, a standard curve of one of the species in triplicate and 24 single-cell samples, with eight samples per species, also in triplicate. The qPCR reactions used 96-well plates with SYBR Green master mixes that were prepared under a UV hood and sterilized by UV light for 20 min. The master mix for each reaction was composed of 10 µl of blue buffer, 0.5 µl of yellow buffer, 0.5 µl of each primer, 6.5 µl of RNA free water and 1 µl of DNA template. The qPCR was performed using the QuantStudio 1 Real-Time PCR thermocycler (Applied Biosystems, Thermo Fisher Scientific) in the following cycling conditions: 95 °C for 2 min at hold stage followed by 40 cycles of 15 s of 95 °C for denaturation, 1 min of 56 °C for annealing and a final Melt Curve Stage of 95 °C for 15 s, 56 °C for 1 min and 95 °C for 1 s. The temperature in the Melt stage increases by 0.15 °C/second. We calculated the mean and standard deviation in the COI copy number of each sample and plotted linear regressions between each pair of curves to assess the congruence of the quantification results (Fig. S1). We observed high congruence results between the standard use (see Results) and chose to amplify all samples using a single standard*.* Each reaction counted with 23 single-cell samples, a negative control, a negative extraction control and a series of standards for one species, all in triplicates.

### Quantitative data analyses

Before downstream analyses, the data were evaluated to exclude potentially inaccurate quantifications. We excluded quantifications where one replicate showed a significant deviation from the two other replicates, and excluded all quantifications with less than 2 copies per 1 µl of DNA extract, to prevent usage of single cell amplification results that are too close to the lower detection limit of the thermocycler. COI quantification data, the volume of the specimens in µm^3^ as well as the SSU quantification data from^17^ are provided in Supplementary Material 2. After assembly of the dataset, we evaluated if a significant relationship exists between the COI copy number and the cell volume for all species analyzed together, and for the species individually using a linear regression associated with a Pearson correlation coefficient. Similarly, we tested for the correlation between COI and SSU gene copy numbers for all species together (Fig. 3B) or individually (Fig. S3).

Next, we evaluated if a significant difference exists in COI copy number between species of foraminifera for individual cells. To choose the correct statistical approach, we ran two Shapiro–Wilk normality tests on raw and logarithmically transformed data and concluded that not all COI gene copy number per unit volume distributions followed a normal distribution. Therefore, we chose the non-parametric Kruskal–Wallis and Wilcox tests for multiple comparisons of species and pairwise comparisons of species, respectively.

Finally, we compared the COI gene copy number per unit volume data with the size of individual cells. We added to the analyses the quantitative data generated by^11^ on 204 specimens belonging to seven species of Larger Benthic Foraminifera (LBF) (*Amphisorus* sp., *Amphistegina lessonii, Baculogypsinoides spinosus, Calcarina spengleri, Heterostegina depressa, Neorotalia gaimardi, Operculina ammonoides*). We calculated the logarithmic correlation coefficients ‘a’ and ‘b’ between the surface area and the biovolume of the MicroCT-scanned specimens for each LBF species, following Eq. 2, using the nonlinear least squares function (nls()) in R.

2 $${\text{Biovolume}}\;({\text{mm}}^{3} ) = {\text{a}}*{\text{Surfacearea}}\,({\text{mm}}^{2} )^{\text{b}}$$

The resulting coefficients, their standard error, and probability can be found in Table 1. In a next step, we measured the surface area of specimens from which DNA was extracted using the pre-extraction shell photographs. Using this value, we calculated the biovolume of the specimens, with the calculated coefficients ‘a’ and ‘b’ (Table 1) using Eq. 2.

**Table 1:** Logarithmic correlation coefficient calculated from the microCT-scanned specimens.

Species	a	b	Standard error a	Standard error b	Probability a	Probability b
*Amphistegina lessonii*	0.05152607	2.0443404	0.006163699	0.11925957	5.709912e−09	4.879374e−16
*Heterostegina depressa*	0.05652766	1.1795028	0.003541088	0.05281369	1.362780e−15	2.202880e−19
*Operculina ammonoides*	0.06095829	0.9218563	0.005973353	0.10332883	9.169620e−11	1.547754e−09
*Calcarina spengleri*	0.06360216	1.3256847	0.003914997	0.11023855	1.837523e−15	2.363555e−12
*Amphisorus sp.*	0.07005834	1.1523716	0.007457344	0.03482399	3.751074e−10	5.423800e−24
*Baculogypsinoides spinosus*	0.11882337	1.3369176	0.002527660	0.04861388	6.448947e−26	3.305610e−20
*Neorotalia gaimardi*	0.13275548	1.4621340	0.010175383	0.08199554	2.020392e−13	8.037457e−17

We provide the data on the number of COI copies, and the volume of individual specimens of LBF as measured in Girard et al.^13^ as Supplementary Material 3. We tested whether a global relationship exists between size and COI copy number in foraminifera when merging planktonic foraminifera and LBF data, and whether the COI gene copy number per unit volume in gene copy number has a relationship with cell size using linear regression with a Pearson correlation coefficient.

All the statistical analyses and plots were conducted using R version 4.3.3^28^ using the packages tidyverse v. 2.0^29^, ggplot2 v. 3.5.1^30^, ape v. 5.7.1^31^, ggpubr v. 0.6.0^32^, openxlsx v. 4.2.5.2^33^, scales v. 1.3.0^34^, ggpol v. 0.0.7^35^, ggpmisc v. 0.6.0^36^, patchwork v. 1.3.0.9000^37^ and provide the code at Github repository: https://github.com/Raph-forams/COI_planktonicForams.

## Results

**Fig. 1:**
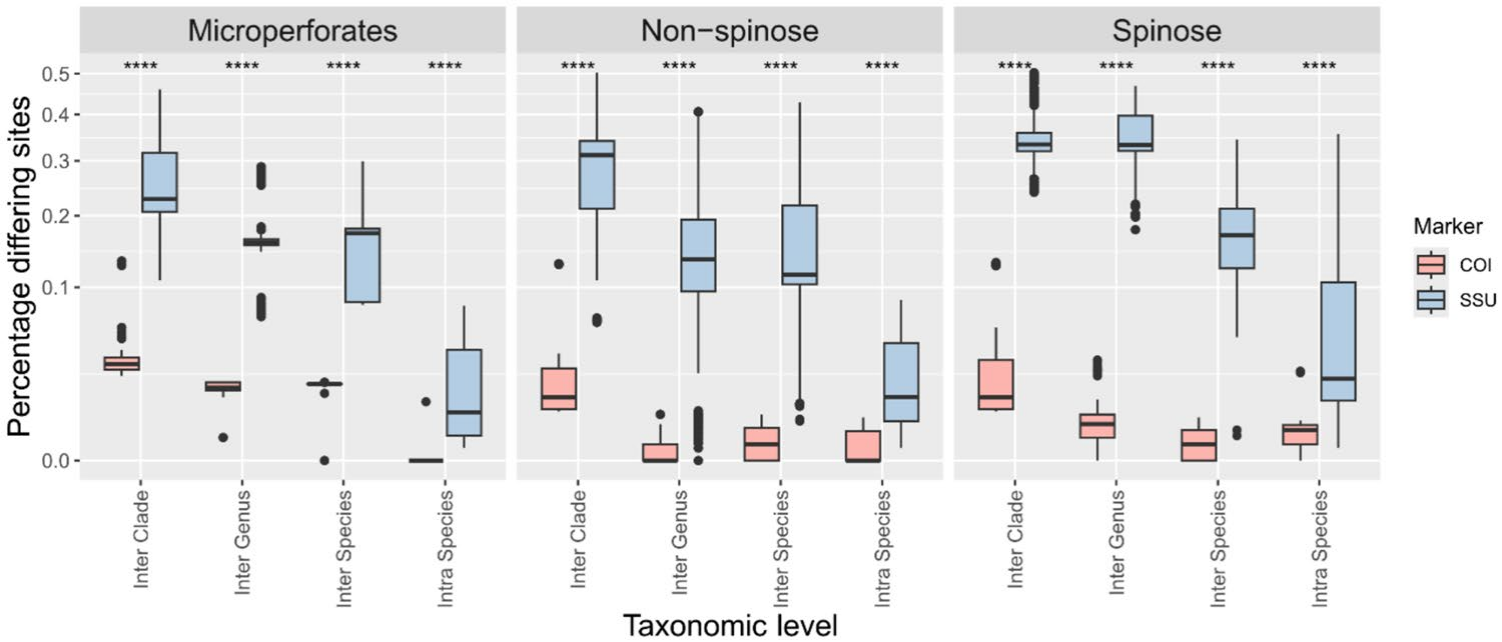
Genetic divergence at increasing taxonomic levels in the SSU and COI gene markers. The stars above the boxplots represent the statistical significance level between the two markers (****p ≤ 0.0001).

**Fig. 2:**
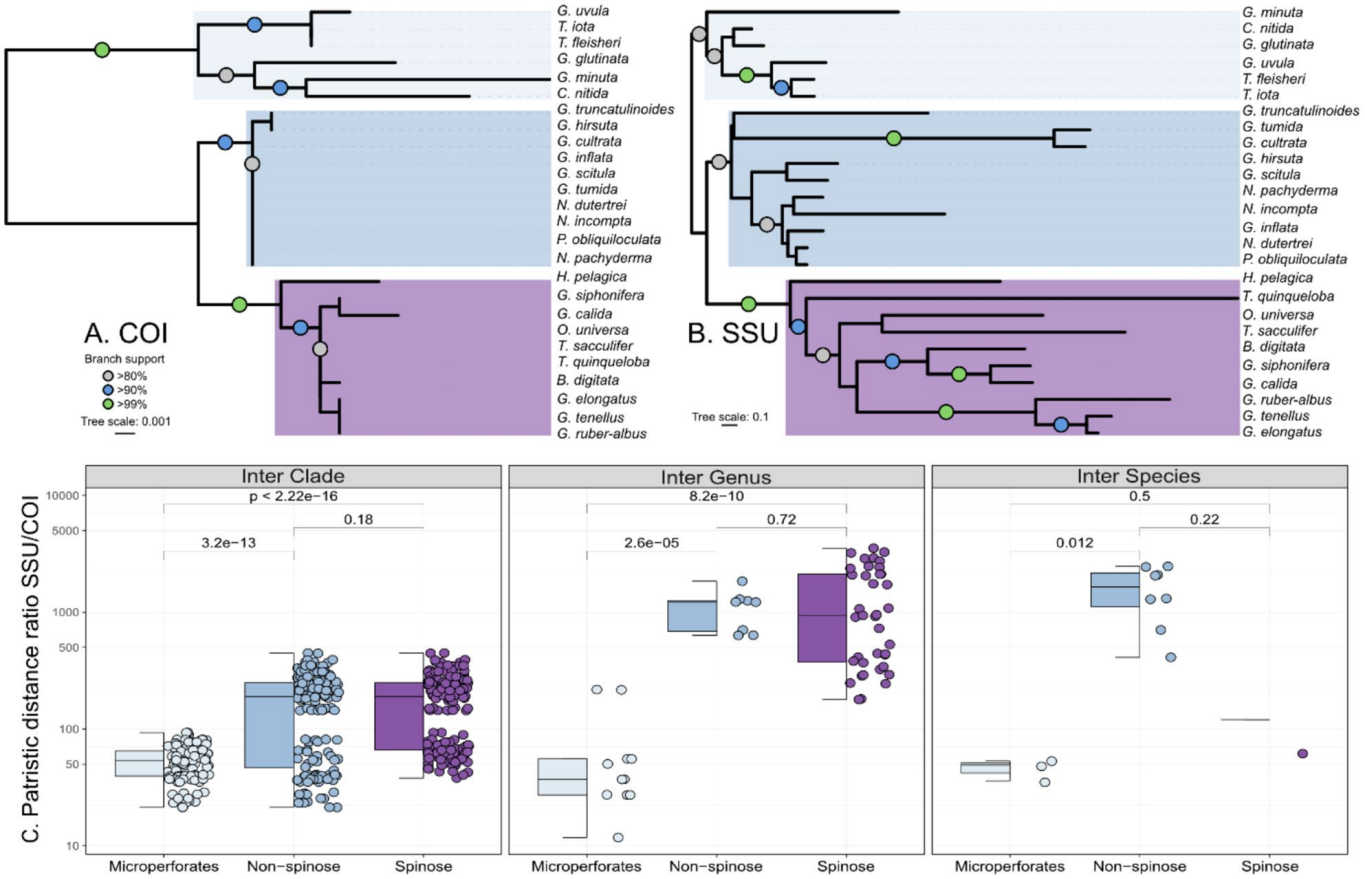
Phylogenetic inference of 26 morphospecies of foraminifera using the COI (**A**) and SSU (**B**) barcodes based on 1000 transfer bootstrap. The colored rectangles indicate the three main clades of planktonic foraminifera: Microperforates (light-blue), Non-spinose (blue) and Spinose (purple). The branch support is indicated by the colored circles on the branches (only above 80%). Note that the COI and SSU trees have different scales (nucleotide substitutions per site) at a ratio of about 1:100. (**C**) Box-plot and jitter-plots showing the ratio in patristic distances (leaf to leaf distance) measured on the trees between the tree clades (Inter-clades), the genus within the clades (Inter-Genus) and the species within the same genus (Inter-Species). The pairwise *t*-test results, are shown above the plots.

We successfully amplified and sequenced the COI barcode of 130 specimens belonging to 26 morphospecies of planktonic foraminifera. The direct comparison between the SSU and COI diversity showed that the COI is conserved and rarely resolves intra-species or sister-species differences (Fig. 1). At increasing taxonomic levels, a consistent genetic divergence appears between different genera and clades for COI, but it remains markedly below the barcode gap of the SSU. We also note that the genetic divergence between successive levels is higher in the Microperforate compared to the two other clades of planktonic foraminifera. The phylogenetic inference confirmed the lack of resolution of the COI marker, although the three main clades of planktonic foraminifera are well supported (> 90% bootstrap Fig. 2A,B). In particular, the Non-spinose species show almost no variability in the COI barcode, except for *G. truncatulinoides* and *G. hirsuta*. For the Spinose clade, the genus *Globigerinoides*, *Globigerinella*, *Beella,* and *Hastigerina* are resolved and all species of the clade Microperforates are resolved except for the sister species of the genus *Tenuitella*. The comparison of evolutionary rates inferred from the phylogenies suggests that the SSU marker gene evolved at least 1000 times faster depending on the clade and taxonomic level considered (Fig. 2C). Even in the Microperforate clade, which has the best taxonomic resolution, COI evolved 25 to 50 times slower than the SSU.

**Fig. 3:**
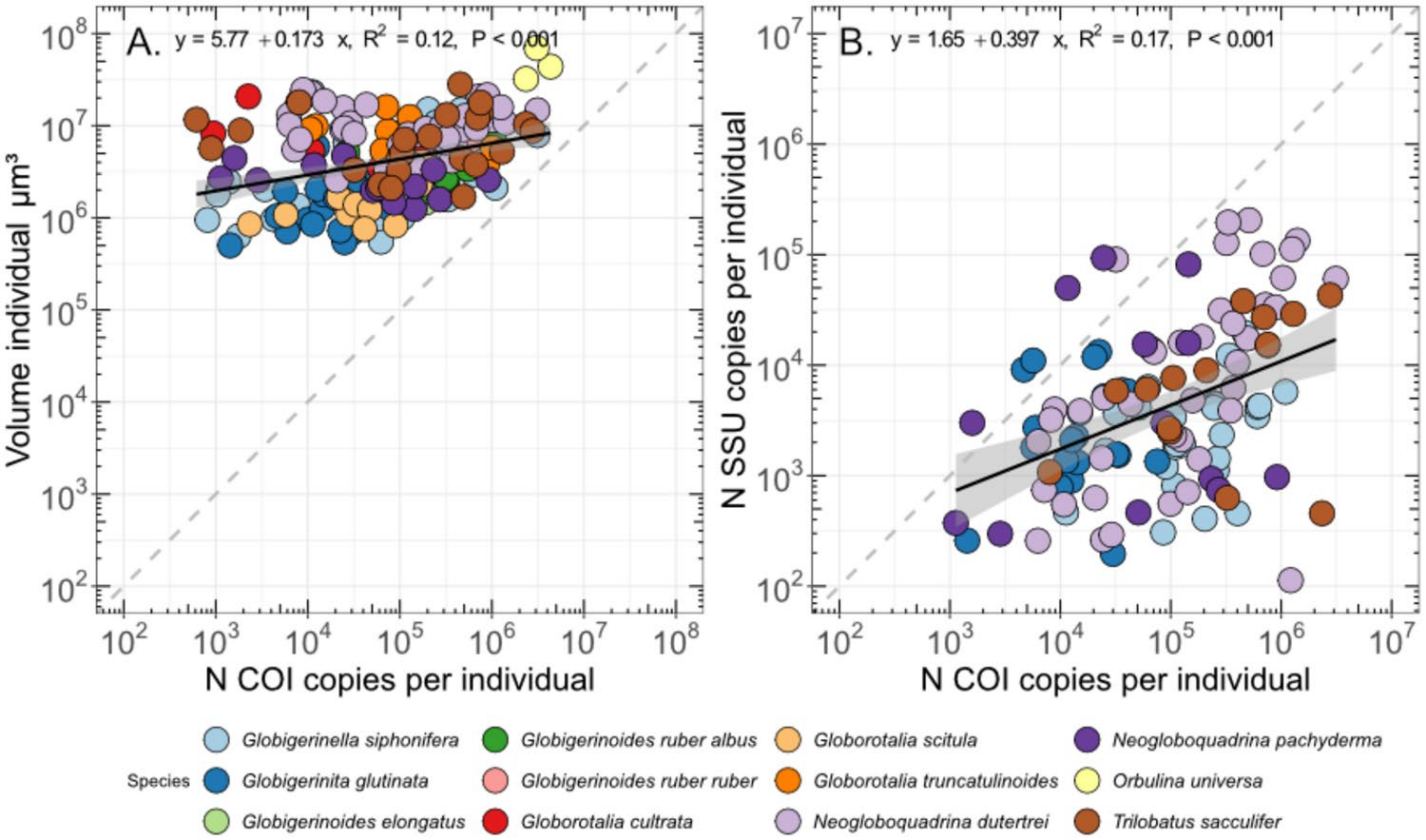
Linear regression between COI copy number and volume of individual foraminifera cell (**A**) and COI and SSU gene copy number (**B**). The linear regression equation, coefficient of determination, and significance level are provided for each graph. Regression for individual species are provided in Figs. S2 and S3.

**Fig. 4:**
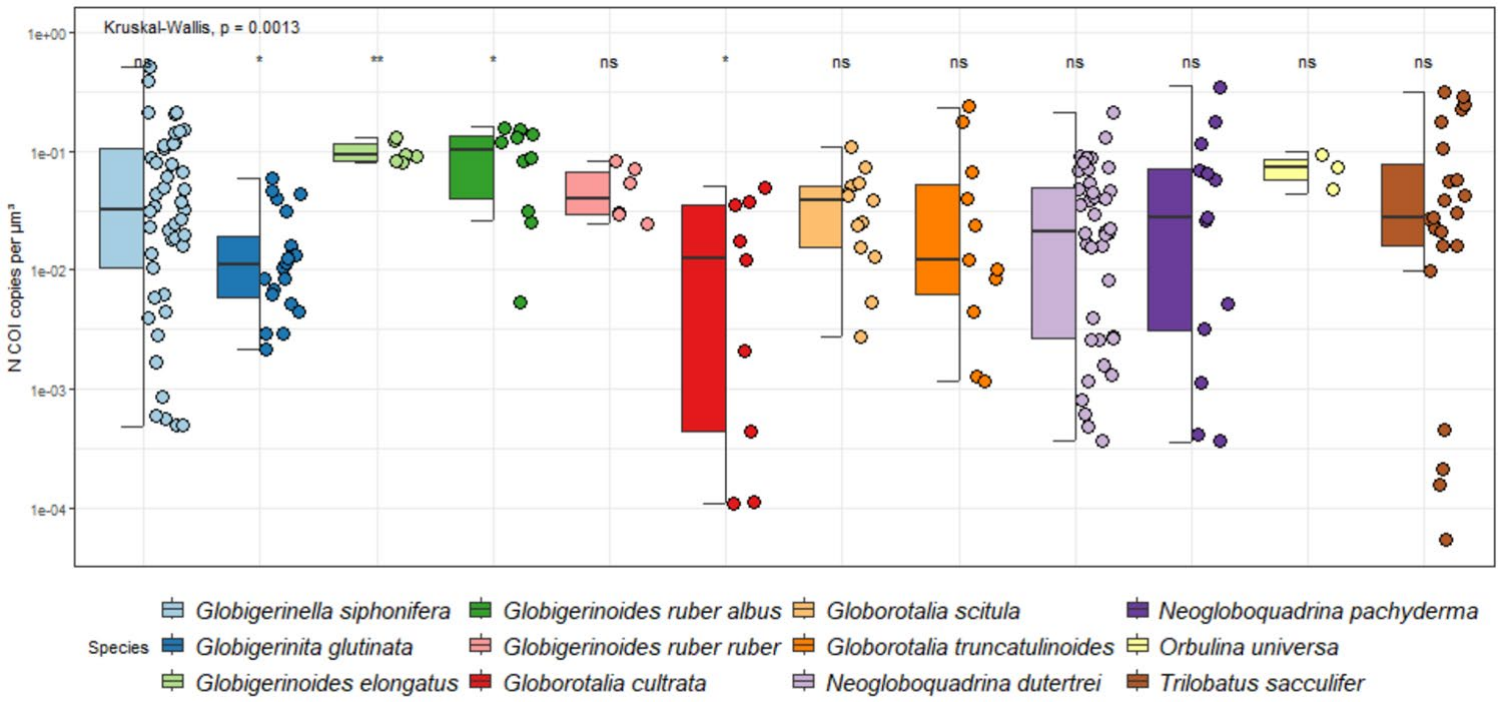
Box-plot and jitter-plot of COI copy number per unit of volume. The *p*-value indicates the Kruskal–Wallis results for the overall comparison and the significance levels for the deviation of individual distributions are shown with stars above the plots (ns = non-significant).

**Fig. 5:**
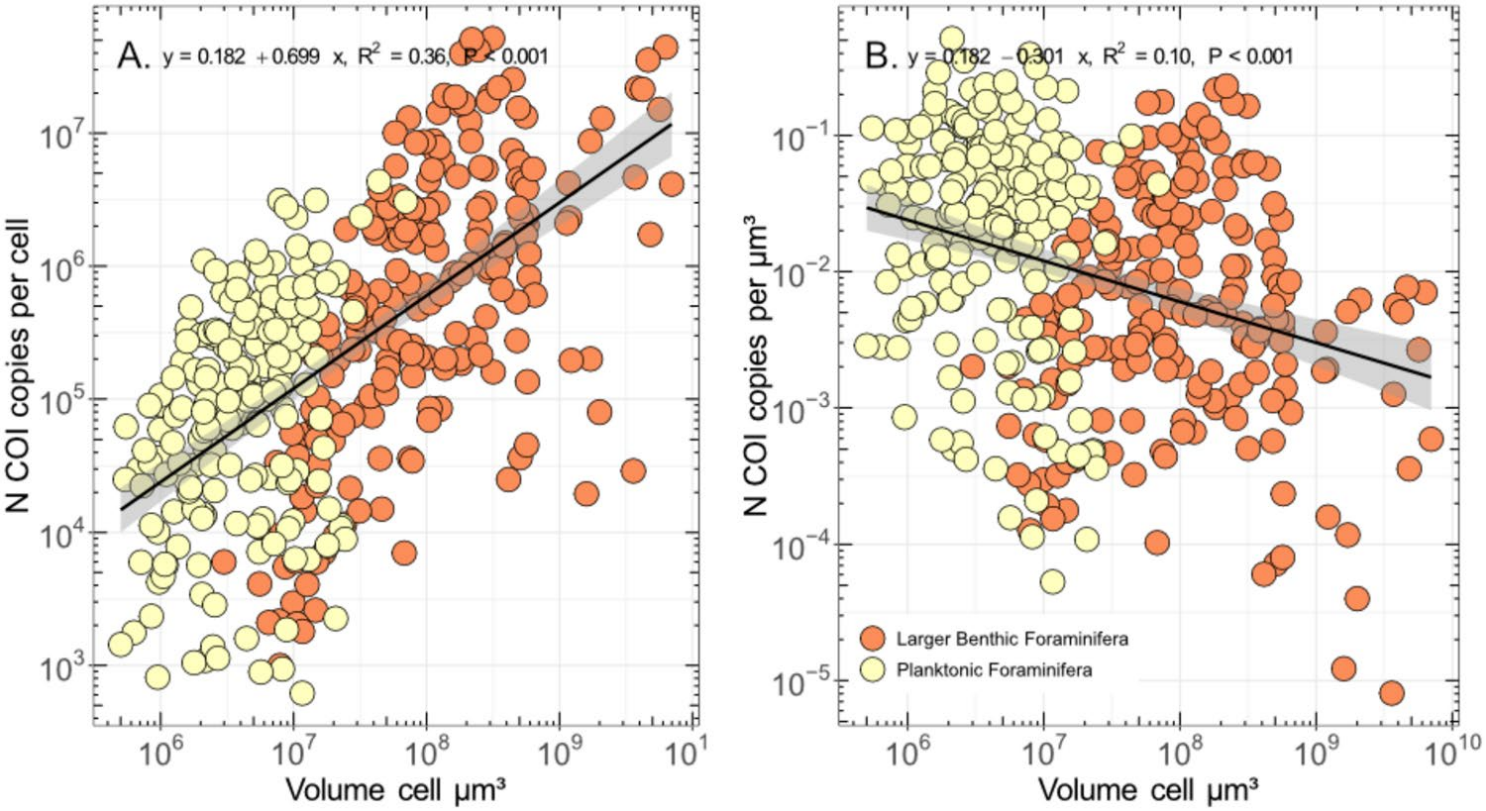
Correlation between number of COI copies and volume (**A**) and COI gene copy number per unit volume and volume (**B**) for planktonic (yellow) and LBF (orange) altogether.

We found an overall weak but statistically significant relationship between cell size and number of COI copy number (Fig. 3A). The scaling of gene copy quantification when using different calibration curves were similar (R^2^ ≥ 0.99; Fig. S1), although the quantification performed with *Trilobatus sacculifer* standard curves returned lower values. Based on these results, we chose to use the calibration curve based on the sequence from *Neogloboquadrina dutertrei* and we could successfully quantify the number of gene copies of 201 specimens belonging to 12 morphospecies^11^. Only three species (*Globigerinella siphonifera*, *Globorotalia scitula,* and *Globigerinoides elongatus*) showed a significant relationship between COI copy number and volume (Fig. S2) next to relationship considering all species (Fig. 3A) and we also observed a weak yet significant overall relationship between SSU and COI copy numbers (Fig. 3B) and similarly only the species *G. siphonifera* showed a significant relationship between SSU and COI copy numbers individually (Fig. S3). Also, the number of COI copies is generally higher than the number of SSU copies, by up to three orders of magnitude (Fig. 3B). When considering the COI gene copy number per unit volume of COI copy number, we observed weakly supported differences between species. However, only four species significantly differ from the average, *G. elongatus* and *G. ruber albus* which have an elevated COI gene copy number per unit volume, and *G. glutinata* and *G. cultrata* which have a lower values (Fig. 4). Finally, the global relationship between number of COI copies and size considering planktonic and larger benthic foraminifera shows a strongly supported relationship (Fig. 5), with a stronger correlation coefficient (R^2^ = 0.36) than when considering the planktonic foraminifera alone (R^2^ = 0.12). Also, we observed a statistically supported negative relationship between the size and COI gene copy number per unit volume (Fig. 5B), indicating that smaller specimens have a higher values compared to larger specimens.

## Discussion

The unusually elevated rate of evolution of the SSU of planktonic foraminifera is known since the earliest studies on these organisms^38–40^ such as it could resolve morphological and even cryptic diversity^41,42^. Our results show that the COI of the planktonic foraminifera does not follow the same trend as it is rather conserved and rarely resolves even morphologically defined species (Figs. 1 and 2), except for species of the Microperforate clade. Initial investigation of the COI resolution on two orders of benthic foraminifera, the Rotaliida, and the Miliolids, indicated that the COI could resolve morphospecies, even those belonging to the same genera^15^. In addition, the direct comparison of the COI and SSU in benthic foraminifera showed a consistently higher intra-genomic and inter-specimen variability in the SSU^10^. Although we did not investigate directly the intra-genomic component in our study we observed no to little sign of intra-genomic variability in the chromatograms of COI sequences, and the specimens of the same species had identical sequences except for *Globigerinita glutinata* (Fig. 1). Therefore, it appears that the COI rate of evolution of planktonic foraminifera may be more consistent with their benthic counterparts, but with clade-specific rates. While the ratio in rate of evolution seems consistent between the Spinose and Non-spinose clades, the Microperforate COI seems to evolve faster (Fig. 2C). Differences in branch length between foraminifera clades are common in SSU molecular phylogenies^43^. However, there is not yet a sufficient coverage of foraminifera diversity based on the COI gene, which would allow to see if the same phenomenon is common for this marker. Yet, the COI appear to have a poor taxonomic resolution and mostly fails to distinguish sister species, and even genus for the macroperforate species of plnaktonic foraminifera.

The slower evolution of the COI gene compared to the SSU gene is intriguing (Fig. 2C), but not necessarily surprising; while the COI marker is a powerful barcode for animal diversity^44^ and is used in various biomonitoring applications^45,46^, it has a lower resolution outside of bilaterian animals^47^ such as cnidarians^48^. The slow-evolving mitochondrial DNA at the base of the Metazoan tree suggests that it is the actual “ancestral state” of animal evolution^49^. For instance, Fungi have limited COI divergence which does not resolve closely related species^50^. In protists, various barcodes have been proposed, such as the ITS-1 or 2, specific regions of the 28S rDNA, or the chloroplastic rbcL and 23S rRNA genes^51^. Despite the variation in rate of evolution between COI and SSU, the basic structure of both trees is identical as the COI resolves the three main clades of planktonic foraminifera with even higher branch support than with the SSU because of the absence of long branches in the COI inference (Fig. 2). Hence, the full mitochondrial genome of planktonic foraminifera may hold a robust signal that could be the key to resolving the evolutionary history of the clades at the generic level or higher, which is not feasible with the SSU because of the length polymorphisms and variation in the rates of evolution between taxa^52^.

The weak but statistically robust relationship between cell size and COI copy number (Fig. 3A) is consistent with the finding from Girard et al. (2024), who evidenced significant relationship between COI copy number and size for seven species of LBF, although we only identified a statistically supported relationship for *G. siphonifera*, *G. elongatus*, *G. scitula* (Fig. S2)*.* The absence of a relationship for the remaining species could be due to a low number of samples or a cell size range too narrow to capture a signal. We also identified a relationship between SSU and COI copy number (Fig. 3B), but that is primarily driven by *N. dutertrei* (Fig. S3). The number of SSU copies is highly variable in foraminifera^17^ and can be explained by the dynamics of the foraminifera genome as exemplified by the monothalameous species *Allogromia laticollaris* that can endoreplicate up to 12,000 times its haploid genome size throughout its life cycle^53^. Explaining the correlation between the COI and SSU gene copy number for a single species of planktonic foraminifera is difficult because we could not identify any mechanisms or processes that could link both. However, we observed that the COI copy number is almost always more abundant than the SSU, up to 1000 times more in a single individual (Fig. 3B). This probably explains why the amplification success rate is about twice as high with the COI than the SSU marker^10^. When considering the COI gene copy number per unit volume, we found that only two species had a higher-than-average gene copy number, *G. elongatus* and *G. ruber albus* while two species had lower than average values, *G. glutinata* and *G. cultrata*. Since we used light microscopy imaging to quantify the size of the foraminifera, we have no information about the ratio between calcite and the total volume of each individual, which can vary between 22 and 42%^54^, and could influence the COI gene copy number per unit volume. Differences in growth rate may also be an explanation for the difference in gene copy number between species but there are no available comparative measurements for all species studied here. Despite the small species-specific variation in COI gene copy number per unit volume, we identified a solid correlation between size and number of gene copies when merging the data for planktonic foraminifera and larger benthic foraminifera, even stronger than when considering the planktonic foraminifera alone (Fig. 5A). This could be due to the methodological difference in data acquisition for both dataset, where the LBF COI copy number were obtained with droplet digital PCR which does not require establishing standard curve, and the volume with CT-scanning^13^, while the planktonic foraminifera data were obtained with qPCR and Keyence light microscopy. We see no obvious reason any method would systematically over or underestimate quantifications results and we observed a smooth transition between the planktonic foraminifera and LBF data clouds in the size range 10^6.5^–10^7.5^ μm^3^ where the largest planktonic foraminifera and smallest LBF species have similar values. With the combined dataset, the size range covers five orders of magnitude and suggests that the relationship could be applied to all foraminifera. While most species-specific calibration curves have been developed on specimens belonging to the Globothalamea clade, a Tubothalamea species of the genus *Amphisorus*^20^. Therefore, only the organic-walled Monothalamea would need to be investigated to confirm if a global correlation between biovolume and COI copy number could be valid for foraminifera. We also noted a slight negative relationship between the size and density of gene copies of COI (Fig. 5B) that could be ascribed to allometric scaling of metabolic rate, which stipulates that total mitochondrial oxygen consumption is lower with increasing organismal size^55^. Therefore, small species of foraminifera will tend to be overrepresented in the metabarcoding studies based on the COI. To which degree this may distort the inferred proportionality from metabarcoding datasets still needs to be tested.

## Conclusion

Our study confirms the scaling between size and COI copy number in in foraminifera, and a remarkable congruence between benthic and planktonic species. However, the COI marker evolves slowly and does not distinguish between closely related species of planktonic foraminifera and would therefore only permit a crude inventory of the main taxonomic groups present in a sample. Therefore, we propose that the COI and SSU barcodes should be used as complementary indicators of foraminifera communities. The COI has the potential to provide robust quantitative information about the abundance of clades or genera of foraminifera, while the SSU has the potential to accurately identify the species diversity in the same samples.

## Supplementary Information

Supplementary Information 1.

Supplementary Information 2.

Supplementary Information 3.

Supplementary Information 4.

Supplementary Information 5.

Supplementary Information 6.

Supplementary Information 7.

## Data Availability

The sanger sequences and associated medatada are available at NCBI under the accession numbers PQ626421-PQ626432 and PQ676554-PQ676671 and are available in the Supplementary Material 1, the qPCR quantification data have been deposited at Zenodo (10.5281/zenodo.14285737) and are available as Supplementary Materials 2 and 3. The R script is available at https://github.com/Raph-forams/COI_planktonicForams.
