# Supplementary material for: Exploring the potential of the COI gene marker for DNA barcoding of planktonic foraminifera
Source: Sci Rep. 2025 Jun 1;15:19205. doi: 10.1038/s41598-025-03842-7 (PMC12127474; doi:10.1038/s41598-025-03842-7)
Supplement: Supplementary file 1 — Supplementary Information 1. [file 41598_2025_3842_MOESM1_ESM.docx]

**Supplementary figure captions**

**Fig. S1.** Linear regression between the results obtained using the calibration curves based on *G. glutinata*, *T. sacculifer,* and *N. dutertrei* COI PCR products.

**Fig. S2.** Linear regression between COI copy number and volume of individual cell for individual species of planktonic foraminifera. The Linear regression equation, coefficient of determination, and significance level are provided for each graph.

**Fig. S3.** Linear regression between COI copy number and SSU gene copy number of individual cell for individual species of planktonic foraminifera. The Linear regression equation, coefficient of determination and significance level are provided for each graph.

**Supplementary material captions**

**Supplementary Material 1.** COI barcode library with collection details, taxonomic attribution, and Sanger sequences of planktonic foraminifera.

**Supplementary Material 2.** COI single-cell qPCR quantification library with collection details, taxonomic attribution, size, and SSU quantification of planktonic foraminifera.

**Supplementary Material 3.** COI single-cell qPCR quantification of larger benthic foraminifera from Girard et al. (2024).
