# Supplementary figures and images for: Exploring the potential of the COI gene marker for DNA barcoding of planktonic foraminifera
Source: Sci Rep. 2025 Jun 1;15:19205. doi: 10.1038/s41598-025-03842-7 (PMC12127474; doi:10.1038/s41598-025-03842-7)

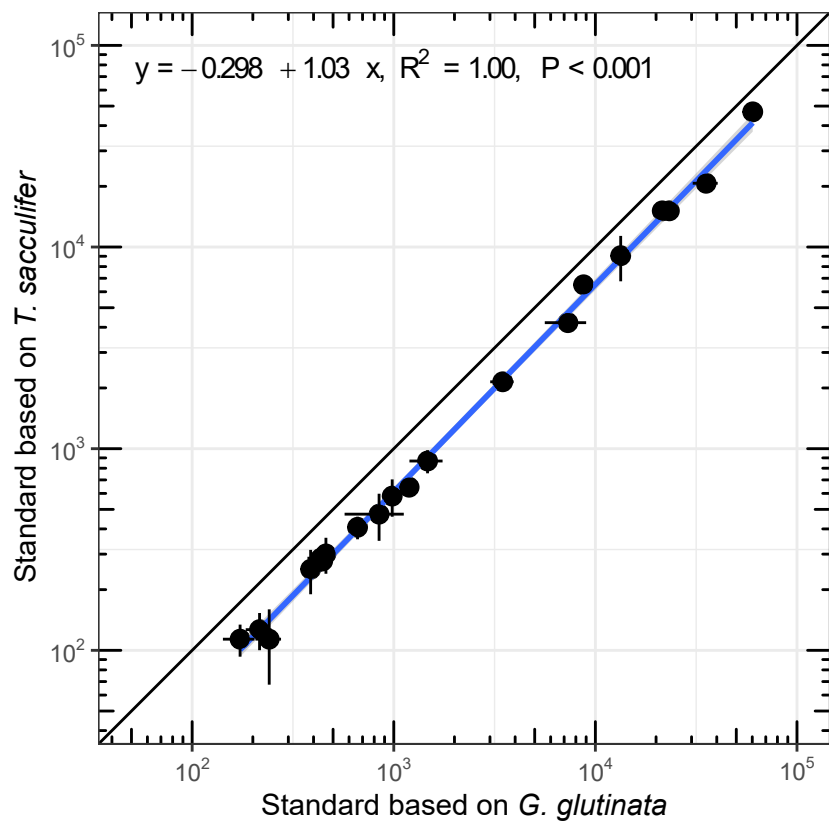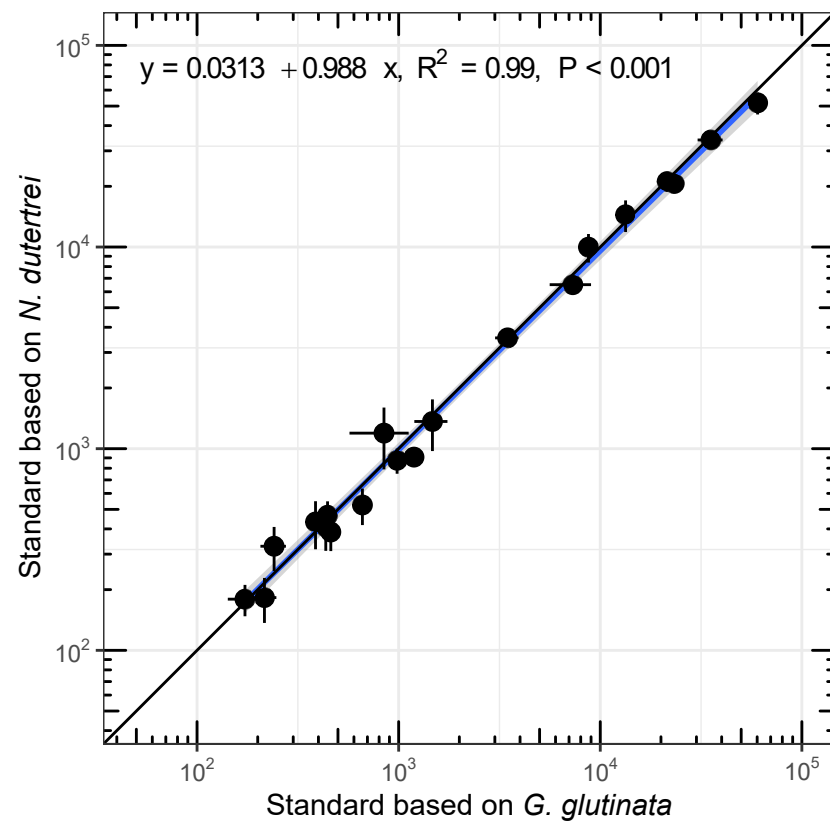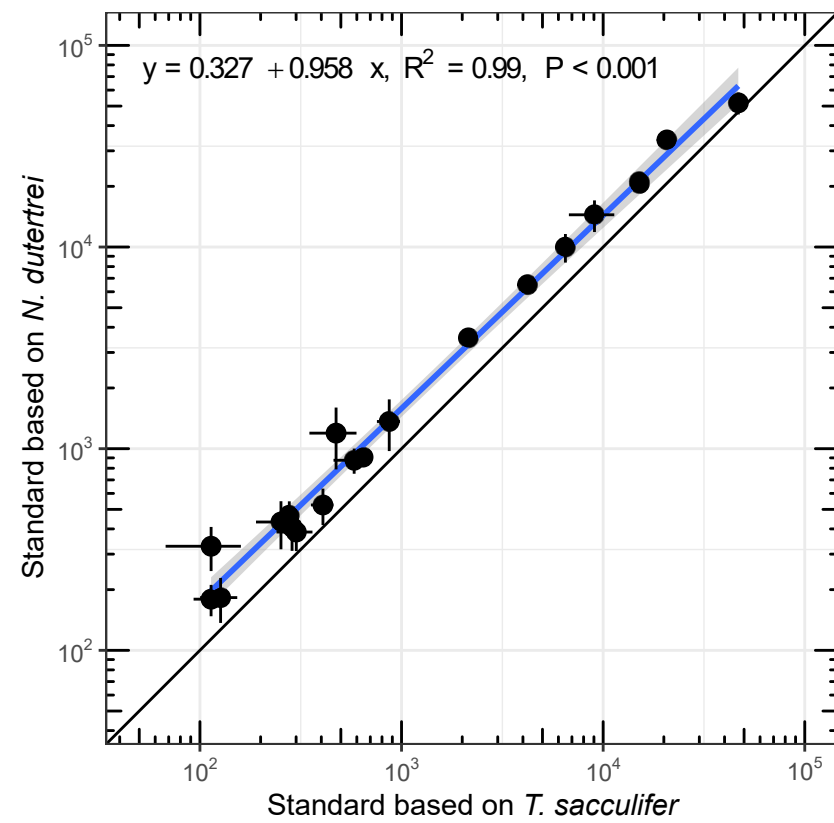

Supplement: Supplementary file 2 — Supplementary Information 2. [file 41598_2025_3842_MOESM2_ESM.pdf]

Volume individual  $\mu\text{m}^3$

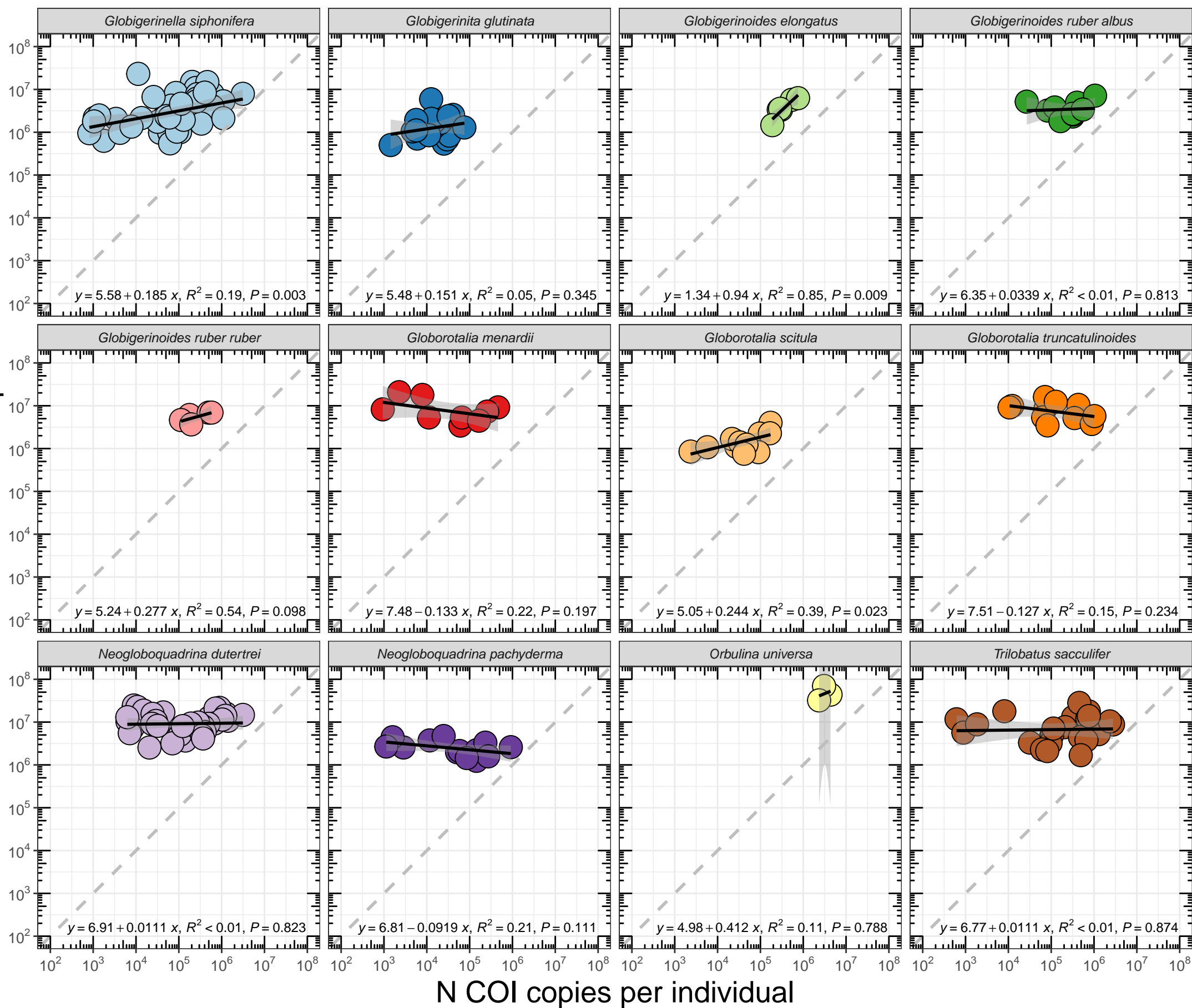

Supplement: Supplementary file 3 — Supplementary Information 3. [file 41598_2025_3842_MOESM3_ESM.pdf]

Volume individual  $\mu\text{m}^3$

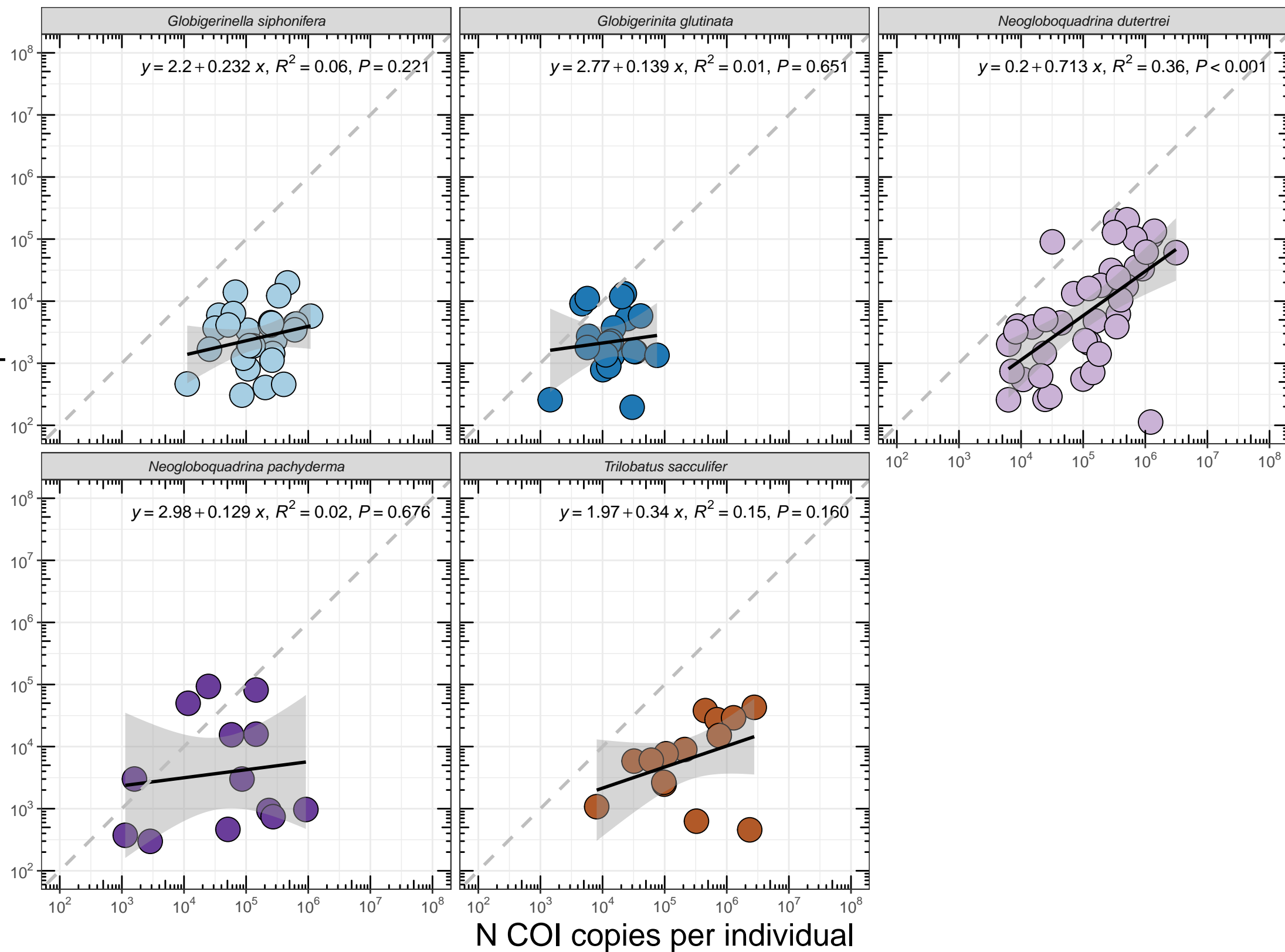

Supplement: Supplementary file 4 — Supplementary Information 4. [file 41598_2025_3842_MOESM4_ESM.pdf]
